# Ascarid infection in wild Amur tigers (*Panthera tigris altaica*) in China

**DOI:** 10.1186/s12917-020-02296-5

**Published:** 2020-03-10

**Authors:** Zhi-wei Peng, Yao Ning, Dan Liu, Ying Sun, Li-xin Wang, Qi-an Zhai, Zhi-jun Hou, Hong-liang Chai, Guang-shun Jiang

**Affiliations:** 1 College of Wildlife and Protected Area, Northeast Forestry University, Harbin, China; 2 Amur Tiger Pk, Harbin, Heilongjiang China

**Keywords:** Wild Amur tiger, *Toxocara cati*, *Toxascaris leonina*, Infection rate, Infection frequency

## Abstract

**Background:**

Wild Amur tigers are a sparsely populated species, and the conservation of this species is of great concern worldwide, but as an important health risk factor, parasite infection in them is not fully understanding.

**Results:**

In this study, sixty-two faecal samples were collected to investigate the frequency and infection intensity of *Toxocara cati* and *Toxascaris leonina* in wild Amur tigers. The *T. cati* and *T. leonina* eggs were preliminary identified by microscopy, and confirmed by molecular techniques. Infection intensity was determined by the modified McMaster technique. Phylogenetic trees demonstrated that *T. cati* of wild Amur tiger had a closer relationship with which of other wild felines than that of domestic cats. *T. leonina* of Amur tiger and other felines clustered into one clade, showing a closer relationship than canines. The average frequency of *T. cati* was 77.42% (48/62), and the frequency in 2016 (100%) were higher than those in 2013 (*P* = 0.051, < 0.1; 66.6%) and 2014 (*P* = 0.079, < 0.1; 72.2%). The infection intensity of *T. cati* ranged from 316.6 n/g to 1084.1 n/g. For *T. leonina*, only three samples presented eggs when the saturated sodium chloride floating method was performed, indicating that the frequency is 4.83% (3/62). Unfortunately, the egg number in faecal smears is lower than the detective limitation, so the infection intensity of *T. leonina* is missed.

**Conclusions:**

This study demonstrated that ascarids are broadly prevalent, and *T. cati* is a dominant parasite species in the wild Amur tiger population.

## Background

The Amur tiger (*Panthera tigris altaica*), also named as Siberian tiger, is a flagship species, which was distributed across the boarders of north-eastern China, the northern part of Korean Peninsula, and the southern part of the Russian Far East [1, 2]. Amur tigers are sanctioned China I level protected animals and are included in the Convention on International Trade in Endangered Species of Wild Fauna and Flora (CITES) Appendix I (https://www.cites.org/eng/app/appendices.php) [3]. Fewer than 400 wild Amur tigers remain in Northeastern Asia, where they are primarily confined to the provinces of Primorye and Khabarovsk (the Russian Far East region) [4]. There are less than 20 Amur tigers distributed within the Changbaishan and Wandashan mountains of Northeast China [5].

*Toxocara cati* and *Toxascaris leonina* are common gastrointestinal parasites of cats [6, 7], both species may affect host fitness and even impair host health [8, 9]. Infection of *Toxascaris* is generally with considerably lower prevalence compared to *Toxocara* infections in domestic cats [10]. The prevalence of *T. leonina* was 5.9 to 30% in domestic cats [11–13], and the prevalence of *T. cati* was 7.2 to 83.3% has been showed in earlier studies [14–16]. However, the situation in captive tiger populations is contrary to that of the domestic cat, a higher prevalence of *T. leonina* than *T. cati* was reported [17, 18]. Ascarids infections have been reported in 3 fecal samples from wild Amur tiger [19]. To increase the knowledge of ascarid frequency in the wild tiger population, faecal samples were collected over a 5-year period in Northeast China and the infection frequency and intensity were analysed in the present study.

## Results

The *T. cati* and *T. leonina* were recognized according to the morphological and molecular characteristics of the eggs present in wild Amur tiger faecal samples. The sequences have been deposited in GenBank under the accession numbers MK381263 (*T. cati*) and MK381264 (*T. leonina*).

The frequency of *T. cati* ranged from 66.6 to 100% in 5 years, and the average frequency was 77.42% (95% CI: 65.59–86.04%) (Table 1). The frequency in 2016 was higher than those in 2013 (*P* = 0.051, < 0.1) and 2014 (*P* = 0.079, < 0.1) (Table 1). The infection intensity of *T. cati* fluctuated over the five-year period from 316.6 n/g to 1084.1 n/g, and the average intensity was 528 n/g (Table 1).

**Table 1:** Comparison of the differences in infection intensity and frequency of *T. cati* from 2012 to 2016

	Total number	Infection number	Average infection intensity (n/g) ± SD	Frequency* (%)	95% Confidence interval (%)
2012	8	7	510.0 ± 903.7	87.5% abc	52.91–97.76
2013	15	10	380.0 ± 783.7	66.6% c	41.71–84.82
2014	18	13	394.4 ± 934.2	72.2% bc	49.13–87.5
2015	12	9	1084.1 ± 1578.4	75% abc	46.77–91.11
2016	9	9	316.6 ± 378.6	100% a	70.09–100
Total	62	48	528.0 ± 1012.0	77.42%	65.59–86.04

Note: * *P* < 0.1; n/g: the number of eggs/gram faeces

For *T. leonina*, only three samples presented eggs when the saturated sodium chloride floating method was performed, indicating that the frequency was 4.83% (3/62). Unfortunately, the egg number in faecal was lower than the detective limitation, so the infection intensity of *T. leonina* was missed.

Within the *T. leonina* internal transcribed spacer 1 (ITS-1) partial nucleotide sequences of four captive tigers (two Amur, one South China tiger, and one Indochinese tiger) and one wild Amur tiger, there were insertion and transversion observed in the South China tiger (*Panthera tigris amoyensis*) and wild Amur tiger, respectively (Table 2).

**Table 2:** Position of insertion or transversion in ITS partial gene sequences of *T.leonina* in Chinese Tiger and wild Amur Tiger respectively

Nucleotide position	Accession Number and Host				
	JF837175 Amur tiger	JF837177 Indochinese tiger	JF837178 South China tiger	MK381264 Amur tiger^a^	Unpublished Amur tiger
24–25	–	–	C	–	–
305	G	G	G	A	G

Note: ^a^ indicated that is wild, others are captured in Zoo

**Fig. 1:**
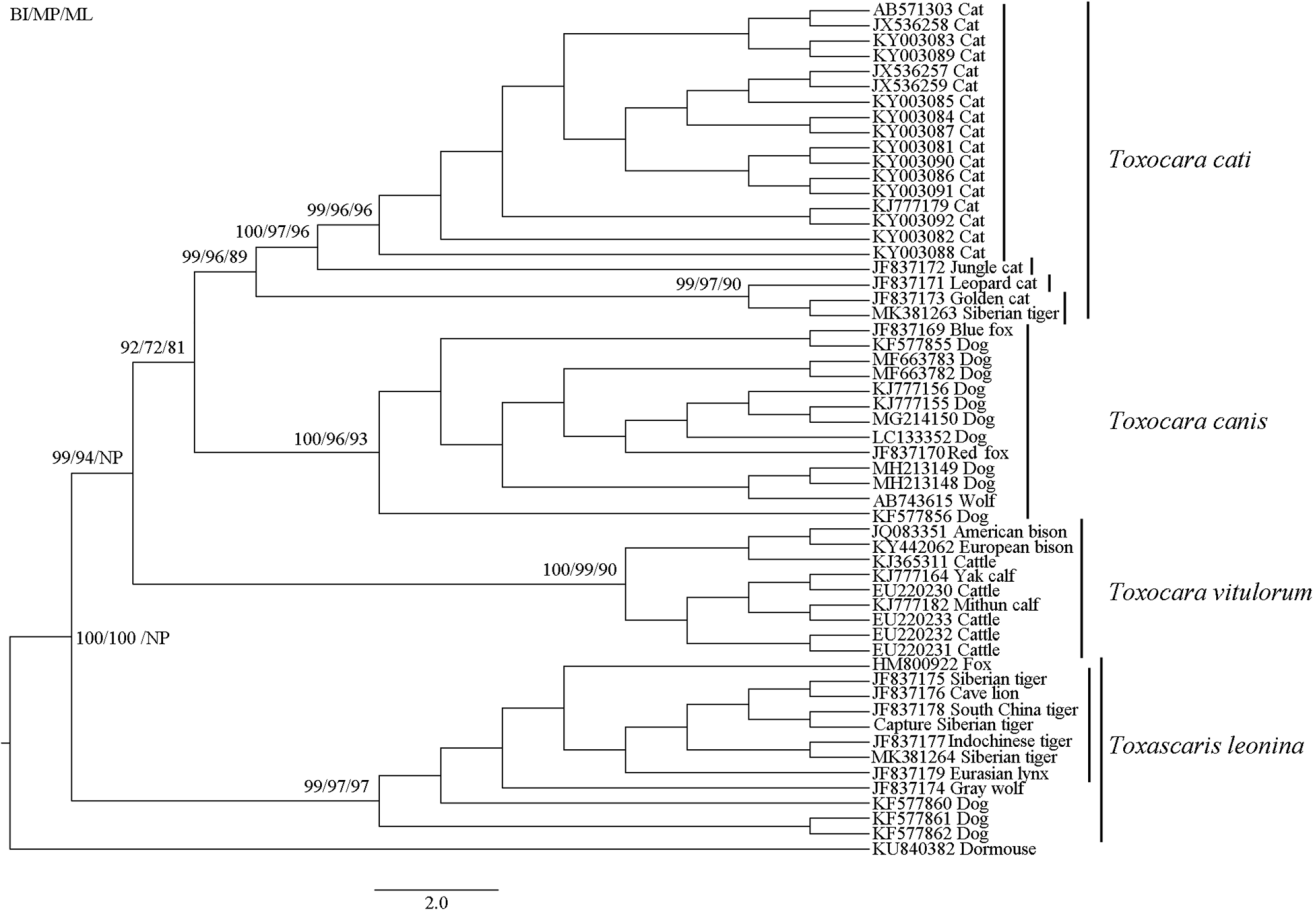
Phylogenetic analysis based on the ITS partial gene sequences of different hosts from *Toxascaris* and *Toxocara*. (NP: not performed)

By comparing ML, MP and BI phylogenetic trees, the results demonstrated that *T. cati* of wild Amur tiger had a closer relationship with which of other wild felines than that of domestic cats. *T. leonina* of Amur tiger and other felines clustered into one clade, showing a closer relationship than canines (Fig. 1).

## Discussion

In the zoo, the Amur had a seriously *T. leonina* (104 n/g, 61.7%) and *T. cati* (56.36 n/g, 49.5%) co-prevalence [17]. In the present study, the infection intensity of *T. leonina* was lower than the detective limitation (60 n/g) and the frequency was 4.83%; but the average infection intensity and the frequency of *T. cati* were 528 n/g and 77.42%, respectively. This illustrated that *T. cati* was only the dominant parasite in the wild Amur tiger population and have a different frequency status comparing with tiger in the zoo. Parasitic infection causes certain effects on immune system [20] and predispose factors for viral infections [21], and also vice versa; viral infections lowering immunity of hosts [21] and the target be liable for severe effects of parasites. As the canine distemper virus has been a risk factor for the Amur tiger [22], co-infection with ascarid will aggravated the health threat. Additional, another situation also needed to be paid an attention. The Amur tiger, a typical solitary carnivore, have a so big habit approximately 400–600 km^2^ [23]; it is hard to understand how *T. cati*, a generally monoxenous nematode, could keep the effective transmission among Amur tigers in the field as in the zoo except the paratenic hosts play a role.

**Fig. 2:**
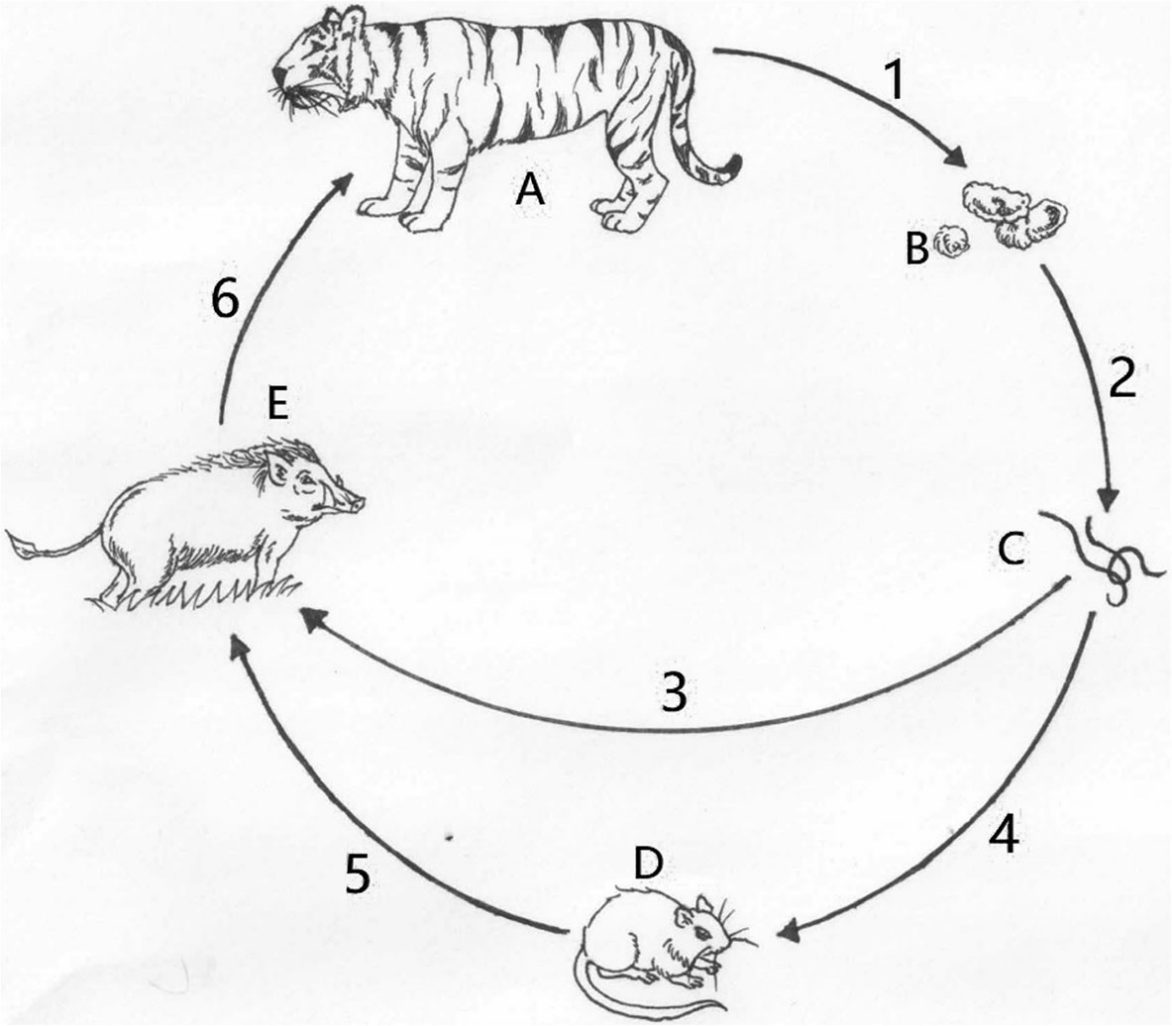
The speculative life history of the *Toxocara cati* in wild Amur tiger. A: Tiger host; B: Eggs in the faecals; C: Earthworm; D: Rodents; E: Wild boar. 1: The eggs of *T.cati* was discharged into environment with faecal of tiger; 2: The eggs of *T.cati* in environment was taken by the earthworm. 3: The earthworm was taken by the wild boar; 4: The earthworm was taken by the rodent; 5: The rodent was taken by the wild boar; 6: The wild boar was preyed by the tiger

Interestingly, wild Amur tiger show a strong preference for wild boar as diet, which were also the most frequently consumed prey [24, 25]. Similarly, the wild boar could take both rodents and earthworm as its diet [26], as well as the earthworm appeared in the diets of the rodent [27]. Besides, the earthworm could carry *T. cati* [28, 29], and *T. cati* appeared in wild boars and rodents served as potential paratenic hosts of *T. cati*, and contributed to persisting in the environment have been found [30, 31]. If the rodent, which taken larva of *T. cati* from earthworm, was preyed by the wild boar, as well as the wild boar, it taken larva of *T. cati* from earthworm or from rodent, was preyed by the tiger, the tiger will be infected by the *T. cati* through this food-chain transmission way (Fig. 2). Then, a hypothesis, *T. cati* transmitted in wild tigers with a special route including paratenic hosts participating was deduced. Actually, the parasites of wild carnivores take the prey as the paratenic host to spread have been showed [32–34], and it is a more reasonable transmission way as the carnivore hosts always have large habits and the oral-faecal transmitting route is impractical in the field. If the speculation we conducted is certified in the future, it will benefit the understanding how *T. cati* spreads in wild big felines efficiently.

The co-evolution between parasites and hosts has been shown in different studies [35–37]. Parasites are generally viewed as having a higher evolutionary potential than their hosts [37, 38]. This may be especially true when parasites have shorter generation times, larger population sizes, higher migration and mutation rates than their hosts [39, 40]. There may be another case between tiger and *T. cati*, as indicated by the results of the present study. Phylogenetic relations among felid species showed that the divergence time (Ma) of the tiger, Asian golden cat, Asian leopard cat, and cat lineages (jungle cat and domestic cat) were 10.78 Ma, 9.43 Ma, 6.18 Ma, and 3.36 Ma, respectively [41]. Interestingly, *T. cati* of the tiger and golden cat diverged first, followed by the leopard cat, jungle cat, and domestic cat (Fig. 1), which coincided with the divergence of these felid hosts. It is supposed that *T. cati* may have evolved into different subspecies or strains to orient to feline host divergence.

## Conclusions

The results of this study demonstrate that *T. cati* is a dominant parasite species in the wild Amur tiger population.

## Methods

### Sample collection and parasites separation

**Fig. 3:**
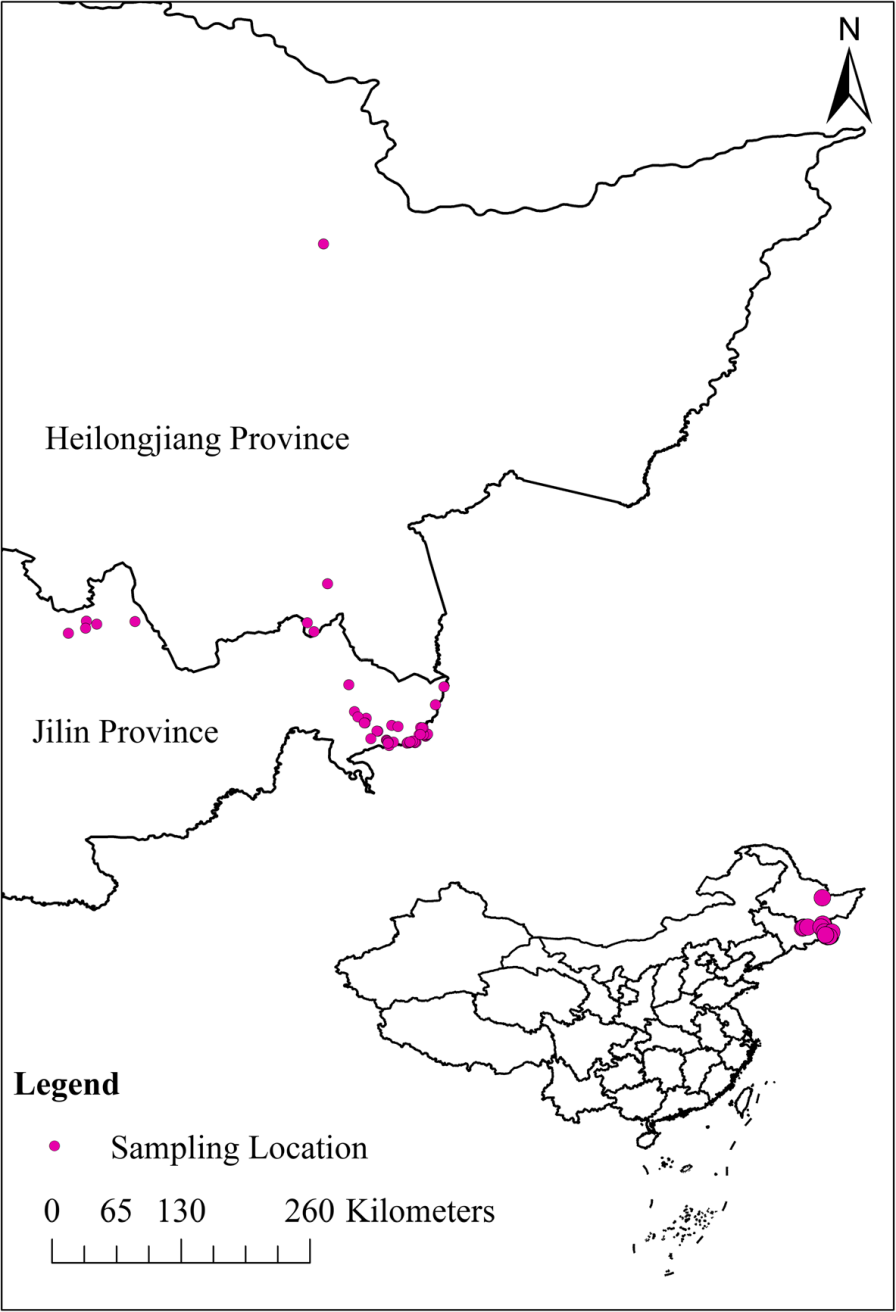
Locations of the Amur tiger faecal sampling sites used in this research

In current work, approved by the local government agents, sixty-two faecal samples of wild Amur tiger were collected during 2012 to 2016 in northeast China from the environment (Fig. 3). Most of them were collected in the Hunchun Amur Tiger National Reserve (HNR), which is a key corridor for movement of Amur tigers among China, Russia and North Korea [24]. Those samples were collected in winter or spring when the project of snow-tracking individual tigers was taken; at that time, the local temperature is absolutely below 0 °C. When samples were brought back to the laboratory, they were stored frozen at − 80 °C. The feces samples were confirmed belonging to tigers were taken by a specific molecular method [2]. The *T. cati* and *T. leonina* eggs were separated with a saturated solution of sodium chloride as the floating medium and identified based on the morphological characteristics [19]. After preliminary identification, the identity of different eggs was confirmed by the polymerase chain reaction (PCR). Infection intensity was determined by the modified McMaster technique [42]. The minimum limit for the infection intensity of *T. cati* and *T. leonina* was 60 n/g.

### DNA extraction and PCR amplification

DNA extraction was performed using a QIAmp DNA Stool Mini Kit (Qiagen Germany) following the manufacturer’s instructions. The partial ITS fragment (727 bp) of *T. cati* was amplified by PCR using a set of primers, including the forward primer FM1: 5′-TTGAGGGGAAATGGGTGAC-3′ and reverse primer FM2: 5′-TGCTGGAGGCCATATCGT-3′ [43]. The partial ITS sequence (452 bp) of *T. leonina* was amplified by universal primers: forward primer S1: 5′-TGCGTTCTTCATCGATCCAC-3′ and reverse primer S2: 5′-AAAGTCTCCAAACGTGCAT-3′. PCR reactions were carried out in a final volume of 25 μl, including 2 mM MgCl_2_, 2.5 mM each dNTP, 10× Takara buffer, 100 pmol each primer, 1.25 U Takara polymerase (Takara), and 1 μL of DNA sample in a thermocycler (BioRad) under the following conditions: initial denaturation at 94 °C for 5 min, 30 cycles at 94 °C for 30 s (denaturation), 60 °C (*T. cati*) or 50 °C (*T. leonina*) for 30 s for annealing, 68 °C for 1 min for extension, followed by a final extension at 72 °C for 8 min. PCR yielded a single band detected in a 1% (w/v) agarose gel upon ethidium bromide staining. PCR products were purified using a Takara minibest agarose gel DNA extraction kit (Takara, Japan) according to the manufacturer’s procedure and sent to Comate Biosciences Co., Ltd. (Changchun, China) for sequencing. For more precision, sequencing of amplicons was performed in both directions (forward and reverse).

### Phylogenetic analysis

The construction of the phylogenetic trees was hypothesized using maximum parsimony (MP), maximum likelihood (ML), and bayesian inference (BI). MP analyses were conducted using PAUP* version 4.0b10 [44]. All characters were weighted equally and unordered, and only potentially phylogenetically informative sites were retained for tree searching. Analyses used a heuristic search with 1000 random stepwise additions followed by tree bisection reconnection (TBR) branch swapping. MP bootstrap branch support values were calculated with 1000 pseudoreplicates with ten random-addition sequences performed in each replication. ML analysis was carried out by MEGA 6.0 with 1000 bootstrap replicates for the estimation of branch support. For BI, the best-fitting models for sequences were HKY + G, which was selected by using Akaike’s information criterion (AIC) as implemented in MODELTEST3.7 [45], and the following settings were applied: 3 million Markov Chain Monte Carlo (MCMC) generations, with a sampling frequency of 100. The first one-fourth generations were discarded as burn-in. The remaining samples were used to generate a majority-rule consensus tree. The frequency of resolving a node was termed a Bayesian posterior probability (BPP). All MCMC runs were repeated twice to confirm a consistent approximation of the posterior parameter distributions. The partial ITS sequence for *Hymenolepis* sp. (GenBank: KU840382) was used as the outgroup.

### Statistical analysis

The old faecal samples collected in the wild environment did not allow the individual animal identification and it cannot exclude that some faecal samples belonged to the same animal. Therefore, this study used the term frequency and not prevalence to describe the proportion of *T. cati* infections in the wild Amur tiger population investigated [46]. The Pnorm function was used to calculate the significant differences in frequency (%), and 95% confidence interval (CI) was calculated in PropCIs function in R version 3.5.2.

## Data Availability

The datasets supporting the findings of this article are included within the article. In wild Amur tiger, GenBank accession numbers of *T.cati* is MK381263 and *T. leonina* is MK381264. For other particular data of *T.cati*, GenBank accession numbers are AB571303, JX536258, KY003083, KY003089, JX536257, JX536259, KY003085, KY003084, KY003087, KY003081, KY003090, KY003086, KY003091, KJ777179, KY003092, KY003082, KY003088, JF837172, JF837171 and JF837173. For other particular data of *T.leonina*, GenBank accession numbers are HM800922, JF837175, JF837176, JF837178, JF837177, JF837179, JF837174, KF577860, KF577861 and KF577862. For *Toxocara canis* and *Toxocara vitulorum*, GenBank accession numbers are shown in Fig. 1.

## Abbreviations

AIC: Akaike’s information criterion
BI: Bayesian inference
BPP: Bayesian posterior probability
CI: Confidence interval
CITES: Convention on International Trade in Endangered Species
HNR: Hunchun Amur Tiger National Reserve
ITS: Internal transcribed spacer
Ma: Megaannus
MCMC: Markov chain monte carlo
ML: Maximum likelihood
MP: Maximum parsimony
PCR: Polymerase chain reaction
*T. cati*: *Toxocara cati*
*T. leonina*: *Toxascaris leonina*
TBR: Tree bisection reconnection
